# The Lymphocytosis of Infection
*A paper read before the Bath and Bristol Branch of the British Medical Association, April 25th, 1928.


**Published:** 1928

**Authors:** T. Wilson-Smith

**Affiliations:** Consulting Physician, Royal United Hospital, Bath.


### THE LYMPHOCYTOSIS OF INFECTION.*

BY

T. Wilson-Smith, M.D., M.R.C.P.,

*Consulting Physician, Royal United Hospital, Bath.*

During the past fifteen years, and especially during the last seven years, considerable interest has centred round the cases which have been variously described under the title of "the lymphocytosis of infection," "infectious mononucleosis," "acute benign lymphoblastosis," and "glandular fever," which though not very common are yet sufficiently frequent to be of importance, both from the theoretical and practical standpoint. I have therefore ventured to bring forward the following case, which was of great interest to me.

Mrs. R. V., æt. 30, residing on the South Coast, had nursed her children with pertussis, and had contracted a bad cough herself, which was possibly pertussis also, for it lasted from April 19th through May, 1927, after which she had been quite well till she went to a ball on July 7th, where she partook of a pasty of some form of meat which she thought must have "turned," for it tasted nasty. She did not, however, have either diarrhœa or vomiting subsequently, and on the next day, July 8th, she bathed in the sea, a pastime of which she was very fond.

On July 9th she was taken with severe headache, which was quite unusual to her, and the headache continued subsequently for a fortnight, with a temperature continuing between 100° and 102°.

* A paper read before the Bath and Bristol Branch of the British Medical Association, April 25th, 1928.

On July 23rd she travelled to Bath, and when I saw her that evening for the first time the auxiliary temperature was 101·4°, and her face was flushed, with a heavy expression strongly suggestive of typhoid. The spleen was enlarged, and could be felt one to two inches below the costal margin, but there were neither spots nor diarrhœa. The liver border was also palpable. All the cervical glands, in the anterior and posterior triangles, including the supraclavicular regions, were enlarged, as also the right axillary and to a lesser extent the left axillary glands. The gums bled at the margins on touching, which she noticed herself when brushing the teeth.

On the morning of the next day, July 24th, I took blood films, the differential count of which showed polymorphs 11–13 per cent. only, lymphocytes 85–89 per cent., eosinophils 4 per cent. A general blood count made the same afternoon showed red blood corpuscles 4,600,000 per c.mm., leucocytes 14,000, so that there was a considerable relative increase, and a smaller absolute increase of lymphocytes.

On July 25th Dr. Odery Symes kindly saw her with me, when blood was taken for agglutination tests against *B. typhosus* and *Paratyphosus A and B*, and it was felt that, if these proved negative, the possibility of acute leukæmia could not be excluded without further watching. The agglutination tests proved negative. Examination of ocular fundi showed neither hemorrhages nor other serious ophthalmoscopic change, nor was any evidence of tubercle found there, or in the lungs, though it may be noted that her sister had died of tubercular meningitis (acute tuberculosis) in infancy. One other point of interest in the history is that I had attended Mrs. V. as a child for a typical attack of glandular fever, but at that time the blood examinations, which would have made the records more complete, were not usually made in such cases. The urine contained a trace of albumen.

That evening, while thinking somewhat anxiously over this case, I recollected having seen a similar case recorded by Dr. A. J. Hall,[1] under the title of "A Case Resembling Acute Lymphatic Leukæmia, Ending in Recovery," and from the details there given we were encouraged to hope that our patient's illness also might come to a favourable issue. I will refer to Dr. Hall's case later.

We thought it well to try and exclude any septic focus, and though a dentist on the South Coast had recently pronounced favourably upon the teeth, I asked Mr. Buchan to see Mrs. V. with me, as there was an upper canine with a sinus near its apex, and one or two lower roots to be seen. Mr. Buchan found that five teeth were dead, and advised their extraction, a few at a time to avoid risk of hemorrhage or severe reaction. After preliminary administration of calcium lactate, extractions were performed on July 29th, August 5th, 15th and September 14th, the first three under gas, the last with gas and ether, a skiagram being finally taken to identify any further septic teeth. The canine, which was root filled, had the largest apical abscess attached to the root which I remember to have seen. All the extracted teeth were unhealthy and several had granulations at the apex. A definite reaction with rise of temperature followed the extraction of the right lower molar, but otherwise Mrs. V. made steady progress towards recovery symptomatically, though a blood film taken on August 1st still showed the percentage of lymphocytes to be as high as 85 per cent.

On September 3rd the total number of leucocytes in the film taken was decidedly smaller, with polymorphonuclears 28 per cent., lymphocytes 67 per cent. Only the tip of the spleen could now be felt on deep palpation, and the glands in the neck were practically normal. The patient looked wonderfully better, and was now just beginning to walk in the garden. On October 5th, 1927, the red blood corpuscles were fully normal or over ; hæmoglobin 75 per cent. ; leucocytes 4,000 per cmm., with polymorphonuclear average 49 per cent., lymphocytes 49·45 per cent. The spleen could no longer be felt, and she returned to her home on the South Coast, and I did not see her again till April 17th, 1928, when she looked in excellent health, and the blood count averaged about 8,000 leucocytes with polymorphonuclears 55 per cent., lymphocytes 42 per cent. There was no enlargement of glands or spleen, and no tendency of the gums to bleed.

While the percentage of lymphocytes is still a trifle above the normal, I think there can be no doubt that this case is not one of leukæmia, but falls under the category of the "lymphocytosis of infection." Let us now look in a little more detail at the recorded cases of the lymphocytosis of infection, taking them chronologically. Osler, writing in 1907,[2] and Dawson Williams in 1907[3] and 1909[4], on Glandular Fever, while describing clearly its symptomatology, make no reference to examination of the blood. The first allusion to this is by some observers in Queensland in 1915,[5] who mention a lymphocytosis occurring, in some cases accompanied by a rash, which possibly may not be of the same nature as those to which we refer.

Dr. A. J. Hall's case, previously mentioned, was that of a gentleman taken ill in March, 1911, with a temperature of 101°, which was followed a few days later by an exudate covering the right tonsil, with enlargement of glands in the right and left sides of the neck, axillæ and groins, enlargement of the spleen and slight albuminuria. The blood examination showed red blood cells 5,400,000, leucocytes 32,000, with polymorphonuclears only 4 per cent., and lymphocytes 96 per cent. Gradual recovery took place, and he was able to go shooting in August, 1911. In 1914 the blood showed still a lymphocytosis of 46·6 per cent.

At the discussion on this case Osler thought that the onset was certainly very suggestive of acute lymphatic leukæmia. Parkes Weber suggested that it was a case of the "lymphocytosis of infection" described by Cabot,[6] and Dr. Hall agreed. Dr. Hall also found a paper by Marchand[7] and concludes: "Both Cabot and Marchand allude to the difficulty of diagnosing these . . . cases from early acute leukæmia until their rapid recovery makes it obvious, and therefore I gather that other observers have fallen into the same pit in times past."

We may now refer to Cabot's and Marchand's papers in abstract:—

*Cabot's Cases.*

1. A post-mortem wound, followed by lymphangitis, adenitis, continued fever, lymphocytosis not over 70 per cent., leucocytes not over 20,000. Slow but complete recovery.

2. A case of boils in an undergraduate, with persistent lymphocytosis of 82 per cent. Total leucocytes 3,400. Recovery.

3. A girl, æt. 20, during a raging epidemic of streptococcal sore throat developed sore throat. Temperature 102°. The cervical glands, lateral and posterior, were the size of marbles, in the right axilla the glands were the size of a hen's egg, the left axillary glands smaller ; spleen and liver were not palpable. There was dullness at each pulmonary apex and over the upper sternal region. Leucocytes 9,000, polymorphonuclears 28 per cent., lymphocytes 71 per cent. Recovery followed.

4. A man æt. 37 stated that he had " a cold " in February, 1912. On March 9th, while at the barber's, he had an attack of vertigo. A troublesome cough continued, and on March 18th there was enlargement of glands in the left side of the neck, the soreness of which passed off in three days. There was slight fever for ten days accompanied by night sweats. The blood count on March 28th showed a leucocytosis of 30,500, with polymorphonuclears 25 per cent. and lymphocytes 75 per cent. On May 18th there was a leucocyte count of 18,800, with polymorphonuclears 57 per cent. and lymphocytes 42 per cent. Recovery followed.

*Marchand's Cases.*

1. Male, æt. 50, typical paratyphoid fever. Polymorphonuclears 43 per cent., lymphocytes 55 per cent.

2. A case of lobar pneumonia, March, 1911. Readmitted November, 1911, with enlarged glands in the neck and at the angles of the jaw. The spleen formed a hard tumour. X-ray examination showed that the thorax was normal. Leucocytes 7,000, lymphocytes 33 per cent. Wassermann test and examination for tubercle bacilli were both negative. The enlarged glands and spleen were still palpable in January, 1912.

4. V. M., æt. 14. This case might now be regarded as one of glandular fever with hemorrhagic nephritis.

5. J. K., November, 1910, æt. 20, was admitted for fever, albuminuria, retinal hemorrhages, enlarged glands in neck, axillæ and groins, slight bronchitis, enlargement of left side of heart with systolic murmur. Pulse 120 to 130, typhoidal state. Blood examination : red corpuscles 1,500,000 per c.mm., hæmoglobin 35 per cent., leucocytes 2,100 per c.mm., lymphocytes 85·5 per cent., polymorphs 6·5 per cent. Death occurred December 12th, 1910.

The diagnosis at first was typhoid, at the end leukæmia or pseudo-leukæmia ; finally, the post-mortem showed streptococcal invasion with punctiform hemorrhages of brain and lungs, and streptococcal foci without suppuration.

Leaving the record of American epidemics recorded by Longcope and others, two papers by H. L. Tidy, the first in association with Morley in 1921 and the second in conjunction with Daniel in 1923, sum up the state of our knowledge of glandular fever, or infectious mononucleosis, while giving an account of an epidemic in a school of thirty boys, of whom twenty-four contracted the disease.

They define it as an acute infectious and contagious disease, principally of but not confined to childhood, characterized by marked enlargement of the cervical glands, and by a less constant and marked enlargement of the spleen, axillary, inguinal and other glands. There may be some tenderness of the cervical glands, but they are not acutely painful : abdominal pain is occasionally severe. The fauces are often reddened, there may be an enlarged tonsil, but exudation is unusual, and changes are slight compared with the marked glandular enlargement. There is no anæmia in the acute stage, but some malaise and enlarged glands by the third day. There may be pyrexia, about 103°, but it may be as high as 105° for a day or two, rarely over 100° for over a week. The glandular enlargement subsides in five to fifteen days ; it may relapse, or, if on one side, pass to the other. Suppuration is extremely rare. The glands may remain palpable for weeks or months, then a prolonged period of depressed health and anæmia follows, before recovery is finally complete.

Hemorrhagic nephritis occurs in 6 per cent. of the cases. The condition has no relationship to mumps, whooping cough, measles, scarlet fever, tonsillitis or to leukæmia, Hodgkin's disease, tuberculosis or pyogenic infections. The incubation is normally from seven to ten days. Epistaxis may occur. The blood examination shows a lymphocytosis which may take time to develop and be transient only.

Tidy goes on to a discussion, and asks the following questions :—

1. "Is glandular fever a clinical entity ?" To which he answers affirmatively.

2. "Are glandular fever and infectious mononucleosis identical ?" To which he replies : "Clinically indistinguishable."

3. Tidy denies that septic infections have any connection with glandular fever. My case appears, however, to show that a connection between the two may exist. The cases of Cabot and Marchand also point definitely to an infection as a causal element, such as a post-mortem wound, a persistent furunculosis, a streptococcal throat, an attack of paratyphoid, or a fatal attack of septicæmia, to which may be added J. Fitch Landon's[10] cases.

**Case 1.**—A white, unmarried woman, æt. 26, was attacked with a mild pharyngitis in February, 1924, with a temperature of 99·4° on the fourth day of illness, pains and prostration. There were two tender glands below the left angle of the jaw, about the size of peas, and a few shot-like, painless, inguinal glands, but no enlargement of liver or spleen. Landon was called again on the fourteenth day as she had not improved, and found the cervical glands a little larger and more tender and the spleen palpable. To exclude malaria a blood examination was made which showed leucocytes 12,000 per c.mm., of which 86 per cent. were mononuclears, the majority being abnormal, somewhat larger than large lymphocytes, with pale blue cytoplasm and oval lobulated or kidney-shaped nuclei which stained intensely.

On the nineteenth day an eminent medical consultant considered the case to be one of acute lymphatic leukæmia. The throat got much worse, and on the twenty-second day there was typical Vincent's angina, which was confirmed by laboratory examination. There was increase in the size and number of the glands of both sides of the neck, and also of the axilla. Two applications of arsphenamin to the throat were followed by rapid improvement, and on May 9th, 1924, mononuclears had fallen to 58 per cent., and in February, 1925, to 28 per cent., only 3 per cent. being abnormal.

**Case 2.**—A coloured girl, æt. 16 years, admitted May 21st, 1920, complaining of pain in the upper abdomen for eight months. She had had follicular tonsillitis for two days. The urine contained a trace of albumen but no casts. Throat swabs were negative to diphtheria, and both Widal and Wassermann tests were negative, as were also the results of rectal and vaginal examinations. There were no tubercle bacilli in the sputum, but X-ray examination of the chest showed a large node at the hilus of the left lung. Blood examination: leucocytes 9,400 per c.mm., mononuclears 80 to 97 per cent., mostly large with "horseshoe" nuclei, no cytoplasmic granules. Persistent increasing temperature with loss of flesh and strength was followed by death five weeks after admission. Three members of the staff concurred in the diagnosis of acute lymphatic leukæmia. The autopsy showed tuberculous bronchopneumonia of upper lobe of left lung, tuberculous tracheal and bronchial lymphatic glands, and miliary tubercle in liver and spleen. The bone marrow was normal.

Landon comments on these cases, that "many non-leukæmic conditions with distinct lymphocytosis may occur, rarely however so marked as to suggest leukæmia," mentioning in children congenital syphilis, scurvy and rickets and pertussis, and in adults tuberculosis, malaria, hæmophilia, typhoid, pellagra. It may occur after the administration of thyroid extract, tuberculin, arsphenamin and pilocarpin, in tetrachlormethane poisoning, and (as mentioned above) Cabot's and Marchand's cases of streptococcal infection.

Tidy and Daniel[9] state in their paper: "In agreement with Longcope, Sprunt and Evans, and others, we consider that the cells are lymphoid in origin. . . . We regard the cells as slightly immature lymphocytes." Fitch Landon says in his paper[10] that "the cytoplasm of these mononuclear cells is greater in volume, and stains more deeply, than that of normal small lymphocytes. Their nuclei are irregular and frequently notched. Degenerating forms are absent in contrast to early acute lymphatic leukæmia. With oxidase stain these cells do not show granules indicating marrow origin, and hence are believed to be of lymphoid origin."

In the case of my patient, Mrs. R. V., the cells were mostly indistinguishable from lymphocytes, chiefly of the small variety, staining deeply with Leishman's stain, but having a greater number showing notching or indentation of the nucleus than in normal blood. Re-examined on October 6th, 1928, her blood showed over five million red corpuscles per c.mm., and 4,000 leucocytes, 52 per cent. of which were mononuclear cells whose reaction to the oxidase method proved them to be lymphocytes.

### SUMMARY.

A case of continued fever, with enlargement of the lymphatic glands and spleen, and associated with a high lymphocyte count, is described. Recovery ensued after radical treatment of dental sepsis.

Similar cases from the literature are reviewed, and the relation between the lymphocytosis of infection and that of acute lymphatic leukæmia is discussed.

[Since I wrote this paper Dr. Odery Symes has kindly drawn my attention to a statement in the American periodical, *Medicine* (February, 1928, p. 8), to the effect that in over 50 per cent. of cases of dental apical sepsis a lymphocytosis occurs.]

### REFERENCES.

[1] Hall, *Proc. Roy. Soc. Med.*, 1915, viii. 15 (Sect. Med.).

[2] Osler, *Principles and Practice of Medicine*, 10th Edition, 1925, p. 372.

[3] Dawson Williams, *Encyclopædia Medica.*

[4] Dawson Williams, *Allbutt's System of Medicine*, 1906, vol. ii., pt. I., p. 591.

[5] Brein, Priestley and Fielding, *Journ. Trop. Med. and Hygiene*, February, 1915.

[6] Cabot, *Amer. Journ. Med. Sc.*, cxlv., 1913, p. 335.

[7] Marchand, *Deutsch. Archiv. f. Klin. Med.*, 1913.

[8] Tidy and Morley, *Brit. Med. Jour.*, 1921, March 26th.

[9] Tidy and Daniel, *Lancet*, 1923, ii., 9.

[10] Fitch Landon, *Amer. Journ. Med. Sc.*. clxx., 1925, p. 37.

